# Non-Invasive Transcutaneous Afferent Patterned Stimulation Therapy Offers Action Tremor Relief in Parkinson’s Disease

**DOI:** 10.5334/tohm.762

**Published:** 2023-08-23

**Authors:** Salima Brillman, Pravin Khemani, Stuart H. Isaacson, Rajesh Pahwa, Ruta Deshpande, Vivien Zraick, Apoorva Rajagopal, Dhira Khosla, Kathryn H. Rosenbluth

**Affiliations:** 1 Parkinson’s Disease and Movement Disorders Center of Silicon Valley, Palo Alto, CA, US; 2 Swedish Neuroscience Institute, Seattle, WA, US; 3 Parkinson’s Disease and Movement Disorders of Boca Raton, Boca Raton, FL, US; 4 University of Kansas Medical Center, Kansas City, KS, US; 5 Cala Health, Inc., San Mateo, CA, US

**Keywords:** Neuromodulation, Non-invasive, Action Tremor, Parkinson’s Disease, Transcutaneous Afferent Patterned Stimulation

## Abstract

**Background:**

Many patients with Parkinson’s disease (PD) experience action tremor (including postural and kinetic tremors) that impair activities of daily living. Transcutaneous afferent patterned stimulation (TAPS) is a non-invasive neuromodulation therapy that modulates tremorgenic activity at the ventral intermediate nucleus (VIM). Most TAPS evidence evaluated relief of action tremor associated with essential tremor (ET). This study evaluated whether TAPS results in similar relief of action tremor associated with PD.

**Methods:**

Forty PD patients with action tremors were enrolled in a prospective, single-arm, open-label study with four weeks of unsupervised at-home TAPS sessions in the dominant hand twice daily in between supervised TAPS sessions at two telemedicine appointments. The primary endpoint was change in tremor power as measured by the on-board accelerometer before and immediately after a stimulation session. Additional study endpoints included change in Movement Disorder Society-Sponsored Unified Parkinson’s Disease Rating Scale Part III (MDS-UPDRS), change in Bain and Findley Activities of Daily Living (BF-ADL) scale, and clinician and patient global impressions of improvement (CGI-I and PGI-I).

**Results:**

TAPS reduced tremor power by 64% (54%–79%) (median (interquartile range), *p* < 0.001), with 79% of patients experiencing at least 50% reduction. When comparing pre-stimulation scores at visit 1 to post-stimulation scores at visit 2, TAPS improved per-task MDS-UPDRS III ratings of postural and kinetic tremors (0.6 ± 0.5, t(34) = 7.05, *p* < 0.001) and per-task patient-ratings of BF-ADL ADL upper limb motion ratings (0.5 ± 0.5, t(34) = 5.69, *p* < 0.001). Clinicians reported improvement in 78–83% of patients and 75–80% of patients reported improvement. Adverse events, most commonly skin reaction at the stimulation site, occurred in 18% of patients.

**Conclusion:**

Objective, clinician-rated, and patient-rated assessments demonstrated that TAPS provided clinically meaningful relief of action tremor in patients with PD.

## Introduction

Many patients with Parkinson’s Disease (PD) experience tremor that interferes with activities of daily living such as eating, drinking and writing, which can lead to embarrassment and limit social interactions [1,2]. Between 46 and 93% of patients with PD experience action tremor [3,4], defined as postural, kinetic and isometric tremor by the International Parkinson and Movement Disorder Society [5]. Current treatment options for action tremor, including pharmacotherapy and surgical interventions, provide insufficient tremor control for many patients, have intolerable side effects, are contraindicated due to common comorbidities, or are inaccessible due to associated cost and risk [6,7].

Transcutaneous afferent patterned stimulation (TAPS) is a non-invasive neuromodulation therapy applied to the median and radial nerves to reduce action tremor in the treated hand [2,8,9,10,11]. The putative mechanism involves modulating tremorgenic activity at key structures within the tremor network, such as the cerebellum [10] and ventral intermediate nucleus (VIM) of the thalamus [12,13,14]. While chronic stimulation of VIM with deep brain stimulation (DBS) has been used to relieve upper limb motor symptoms in both essential tremor (ET) and PD [15,16], and both ET and PD patients are prescribed propranolol for action tremor control [17], to date TAPS has only been evaluated in patients with ET. Studies demonstrating the efficacy and safety of TAPS in ET include three randomized clinical trials (two completed studies randomized to sham [8,9] and an active study randomized to standard-of-care (clinicaltrials.gov NCT05540626)) as well as two longitudinal home-use studies [11,18]. This study sought to evaluate the use of TAPS in PD patients with action tremor.

## Methods

### Study design

**Figure 1:**
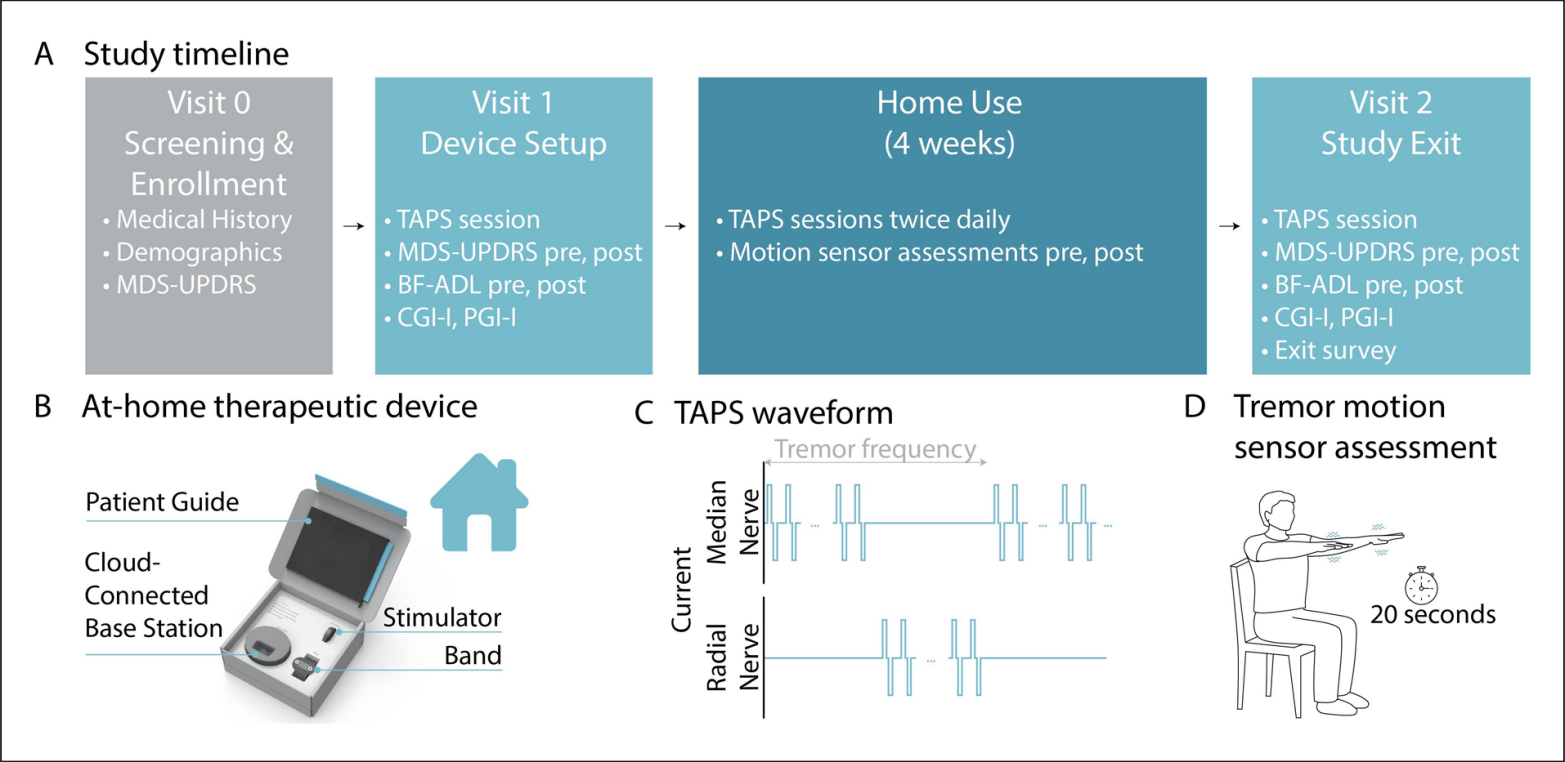
**(A)** The study included three visits and four weeks of home therapy use. **(B)** The TAPS device included a stimulator, band and base station. **(C)** The TAPS device automatically calibrated stimulation to alternate between the patient’s median and radial nerves at the tremor frequency measured by an onboard triaxial accelerometer. **(D)** Patients performed a device-prompted postural hold for calibration and measurements of tremor power during home use.

This was a prospective, single-site, single-arm, open-label study evaluating the efficacy and safety of TAPS in PD patients with action tremors (clinicaltrials.gov NCT05012579). The study was approved by an Institutional Review Board and all patients provided written informed consent. The study consisted of three visits, conducted remotely by telemedicine due to the COVID-19 pandemic, with a four-week home-use period of TAPS between visits (Figure 1A).

Patients were screened and enrolled at visit 0. Patients were eligible for the study if they had a diagnosis of PD, had postural tremor (defined as scoring ≥2 on the Movement Disorder Society Unified PD Rating Scale (MDS-UPDRS) Part III Motor Examination [19] postural tremor task assessment (question 3.15)) in the dominant hand in the medication-off state, had been stable on any PD-related medications for at least 30 days prior to study entry and intended to remain stable for the weeks they would be enrolled in the study, and agreed to withhold the last dose of any dopaminergic medication prior to study visits and report time elapsed since the most recent dose such that the investigator could confirm clinical motor assessment was performed in the medication-off state. Patients were ineligible for the study if they had an implanted electrical medical device (e.g., DBS device, cardiac pacemaker), seizure disorder, skin irritation or wounds at the stimulation site, peripheral neuropathy, presence of other neurodegenerative disorders, botulinum toxin injection for hand tremor within six months of study enrollment, caffeine consumption of more than the equivalent of a cup of coffee (95 mg) within eight hours of study visit, or were pregnant.

Patients were shipped a wrist-worn TAPS device prior to visit 1 (Cala Health, Inc., San Mateo, CA, USA). The TAPS device was commercially available as a prescription therapy for use in patients with ET (K203288). The TAPS device included a band with embedded multi-use electrodes positioned to target the median and radial nerves of the patient’s dominant hand, a removable stimulator, and a base station for stimulator recharging and device data upload (Figure 1B). The TAPS device automatically calibrated stimulation to alternate between the median and radial nerves at the individual’s tremor frequency measured by an onboard triaxial accelerometer [9] (Figure 1C) during a 20-second postural hold prompted by the device (Figure 1D).

At visit 1, a neurologist trained the patient on use of the device and supervised the patient through a 40-minute stimulation session. The neurologist rated six upper-limb motor tasks from MDS-UPDRS Part III Motor Examination [19] before and after the supervised stimulation session, following guidance for assessing these tasks by telemedicine [20]. The six tasks were MDS-UPDRS 3.15 (postural tremor of the hands), 3.16 (kinetic tremor of the hands), 3.17 (rest tremor amplitude), 3.6 (pronation-supination movements of hands), 3.4 (finger tapping), 3.5 (hand movements), each rated on a scale of 0 (“Normal”), 1 (“Slight”), 2 (“Mild”), 3 (“Moderate”), or 4 (“Severe”). The patient also performed eight upper-limb motor tasks from the Bain and Findley Activities of Daily Living (BF-ADL) scale [21] before and after each supervised telemedicine stimulation session with props available at home. Self-rated BF-ADL scores were recorded both before and after the stimulation session. Prior published studies of TAPS for postural and kinetic tremor in ET have utilized patient assessments of BF-ADL tasks using props before and after stimulation sessions [9,11]. Patients were instructed to use the same props for BF-ADL assessment in each remote visit to ensure consistency. The “dial a telephone” task was modified so that patients enter telephone numbers into their cellphones. MDS-UPDRS and BF-ADL ratings were assessed on the treated limb (i.e., dominant limb). The neurologist and patient also rated Clinical and Patient Global Impression of Severity (CGI-S and PGI-S, respectively) at the beginning of each supervised session and Clinical and Patient Global Impression of Improvement (CGI-I and PGI-I, respectively) at the end of each supervised session for every visit [22,23].

Patients were instructed to perform unsupervised TAPS sessions at home twice daily in the dominant hand for 40-minutes for a month. The TAPS device prompted patients to perform a postural hold before and after each session and measured the tremor power using the same triaxial accelerometer used to calibrate the TAPS device. Tremor power has been validated against simultaneously measured MDS-UPDRS ratings [24,25]. Patients who had only re-emergent tremor would receive a score of 0 for postural tremor in MDS-UPDRS III rating at visit 0 [26] and thereby were excluded from the study.

At visit 2, patients performed a final supervised stimulation session with MDS-UPDRS ratings, BF-ADL ratings, CGI ratings, PGI ratings, and an exit survey. Patients were asked about the duration of treatment effect using the following question: “On average, how long did postural hand tremor relief last after a stimulation session? __ minutes __N/A.”

Adverse events (AEs) were self-reported by patients, recorded by study personnel, and monitored by an independent safety reviewer (co-author PK) who is a board-certified neurologist and movement disorder specialist. Percentage of AEs was calculated as the number of reported AEs divided by the number of patients enrolled in the study.

### Study endpoints

The primary endpoint was improvement in tremor power. Wrist-worn accelerometer recordings of postural holds, before and immediately after therapy sessions of 40 minutes were included in the primary endpoint results. Tremor power improvement ratio (TPIR) was calculated as the ratio of tremor power from pre- to post-stimulation postural holds over each valid session in each patient [11]. Subsequently, the median TPIR was computed in each patient across all valid sessions from study visits and home-use phase. A TPIR value of 1 indicated that tremor power was unchanged after the therapy session, TPIR greater than 1 indicated that tremor power improved after stimulation, and TPIR less than 1 indicated that tremor power worsened after stimulation. Valid sessions for TPIR calculations were identified using the following data filter criteria: (1) session duration of at least 20 out of 40 minutes (ensures a sufficient stimulation duration for therapeutic effect [27], (2) availability of both pre- and post-stimulation postural hold measurements, recorded within 15 minutes of each session, (3) latency of at least 120 minutes from the last TAPS session performed (avoids consecutive sessions), and (4) kinematic data free from voluntary motion artifact (suggesting non-adherent postural holds) and hardware recording artifacts (signal saturation). See the supplemental methods for more details about how voluntary movement artifact was assessed. Patients were excluded from the primary endpoint analysis if PD-related medications were changed during the study or they had fewer than ten TAPS sessions with accelerometer recordings not contaminated by motion artifact.

The secondary endpoints were improvement in clinician-ratings of action tremor, defined as the average change per-task in MDS-UPDRS 3.15 (postural tremor of the hands) and MDS-UPDRS 3.16 (kinetic tremor of the hands), and patient-ratings of upper limb activities of daily living, defined as the average change per-task in the eight assessed BF-ADL tasks. Each of these were analyzed in the dominant hand from before the supervised stimulation session in visit 1 to after the stimulation session in visit 2, as well as before to after the stimulation session within each visit. The exploratory endpoints included CGI-I, PGI-I, and improvement and responder rate of individual tasks in MDS-UPDRS III and BF-ADL. Responder rates were defined as percent of patients improved by at least one point following stimulation of whom rated at least mildly impaired in the assessed task (i.e., MDS-UPDRS III or BF-ADL score ≥ 2). Patients whose PD-related medications were changed during the study were excluded from the secondary endpoint analyses.

The order of endpoints for statistical analysis were pre-defined in the statistical analysis plan. Analysis began with the primary endpoint, TPIR, followed by secondary endpoints, MDS-UPDRS and BF-ADL. The primary endpoint was analyzed using one-sample Wilcoxon’s signed-rank test to determine whether TPIR (primary endpoint) was greater than 1 with one-sided *p* value < 0.025 considered significant. The nonparametric Wilcoxon’s signed-rank test was used to account for skewed distribution in the TPIR. For the secondary endpoints, paired sample *t*-tests were performed to compare pre and post TAPS sessions within visits 1 and 2, as well as pre-stimulation of visit 1 to post-stimulation of visit 2. Two-sided *p* value threshold was set to 0.05. Holm-Bonferroni method was used to control for multiple testing. Study data is available upon request.

## Results

The study enrolled 40 patients and 36 patients completed the study (Table 1). Reasons for study withdrawal included device malfunction, dislike of the stimulation sensation, uneasiness around cloud connectivity and data collection, and investigator decision following an AE classified as unlikely to be device related.

**Table 1:** Enrolled patient characteristics.


DEMOGRAPHICS	

**Gender, female**	25% (10/40)

**Age** (years)	67.1 ± 9.9 (41–85)

**Race**	

Asian	15% (6/40)

Black or African American	3% (1/40)

White	83% (33/40)

**Ethnicity**	

Hispanic or Latino	8% (3/40)

Not Hispanic or Latino	88% (35/40)

Unknown or not reported	5% (2/40)

**CLINICAL CHARACTERISTICS^1^**	

**Age at onset of first PD symptom** (years)	60.5 ± 10.0 (38–82)

**Age at onset of hand tremor** (years)	61.1 ± 9.7 (39–82)

**Age diagnosed** (years)	61.9 ± 9.9 (38–82)

**Duration from onset** (years)	6.6 ± 3.8 (1–16)

**Duration from hand onset** (years)	6.0 ± 3.6 (1–16)

**Duration from diagnosis** (years)	5.2 ± 3.1 (1–13)

**On any PD medication**	98% (39/40)

**MDS-UPDRS action tremor** ^2^	1.7 ± 0.5 (1–4)

**MDS-UPDRS treated hand** ^3^	1.7 ± 0.6 (1–4)

**BF-ADL treated hand** ^4^	2.0 ± 0.5 (1–4)


Categorical data reported as percentage (N/40); Continuous data reported as mean ± 1 standard deviation (range). ^1^At enrollment (Visit 0). ^2^Average of MDS-UPDRS postural tremor and kinetic tremor scores (questions 3.15 and 3.16, respectively). ^3^Average of six MDS-UPDRS upper limb tasks (questions 3.4–3.6 and 3.15–3.17 evaluated on treated limb). ^4^Average of eight BF-ADL tasks evaluated on treated limb.

One patient was further excluded due to having fewer than 10 sessions in total. The remaining 35 patients performed 1,764 sessions and 427 sessions were excluded for not meeting the criteria of valid sessions. An additional 64 sessions from 1 patient who had medication changed were also removed from the main analysis. Therefore, 1,273 total home-use therapy sessions from 34 patients were available for analysis of tremor power.

**Figure 2:**
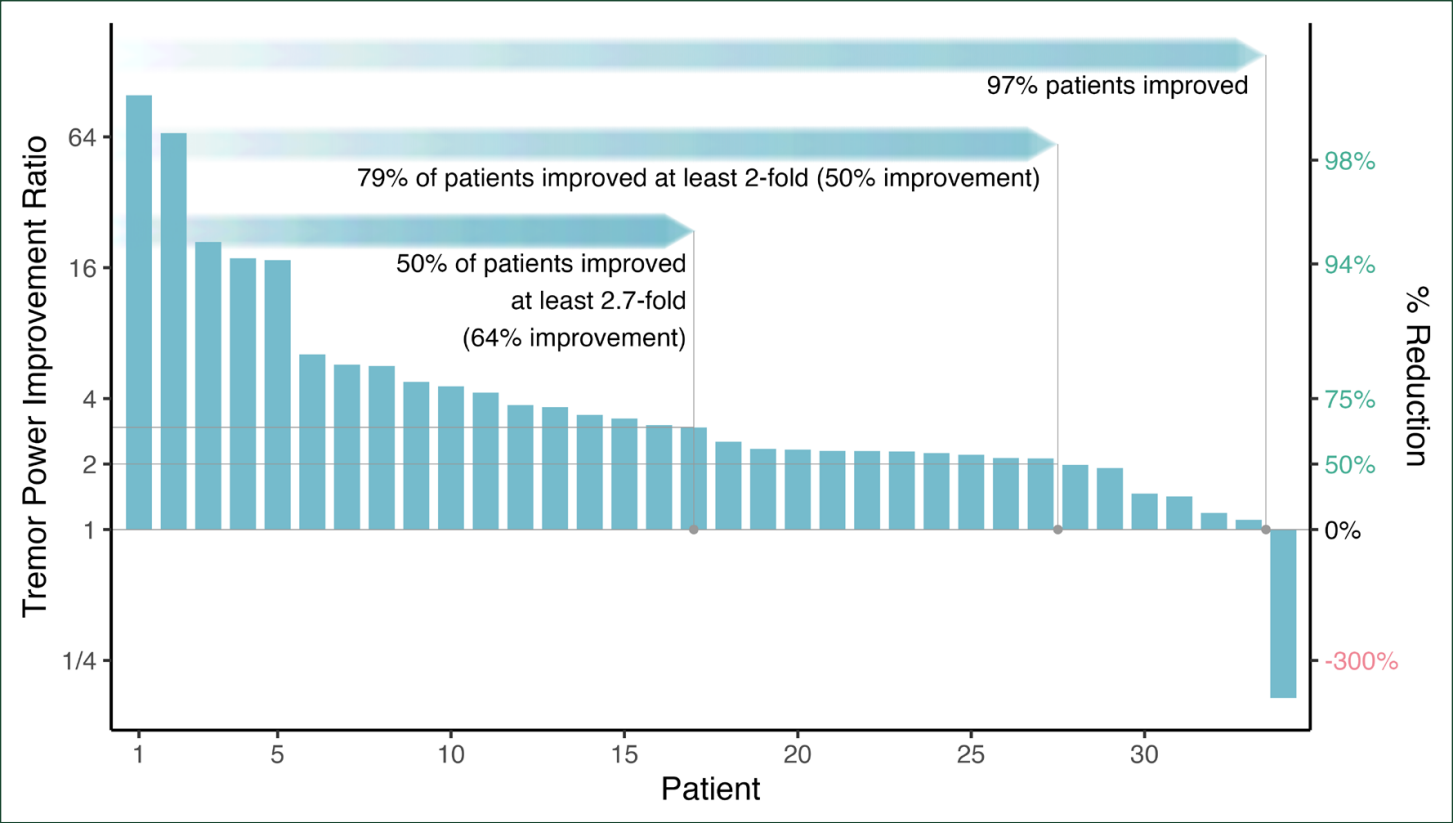
**Tremor improvement as measured by motion sensors (primary endpoint).** TAPS improved tremor during a month of unsupervised home use, with 50% of patients showing at least 2.7-fold improvement (median, 64%; interquartile range, 54%–79%) improvement with 79% of patients improved at least 2-fold and 97% of patients improved.

Tremor power significantly reduced by a median of 64% (interquartile range 54% – 79%) from before to after therapy (median TPIR = 2.74, *Z* = 590, *p* < 0.001, 97.5% lower confidence bound = 2.47), with 79% of patients experiencing 50% or greater tremor reduction and 97% of patients experiencing tremor reduction (Figure 2).

**Figure 3:**
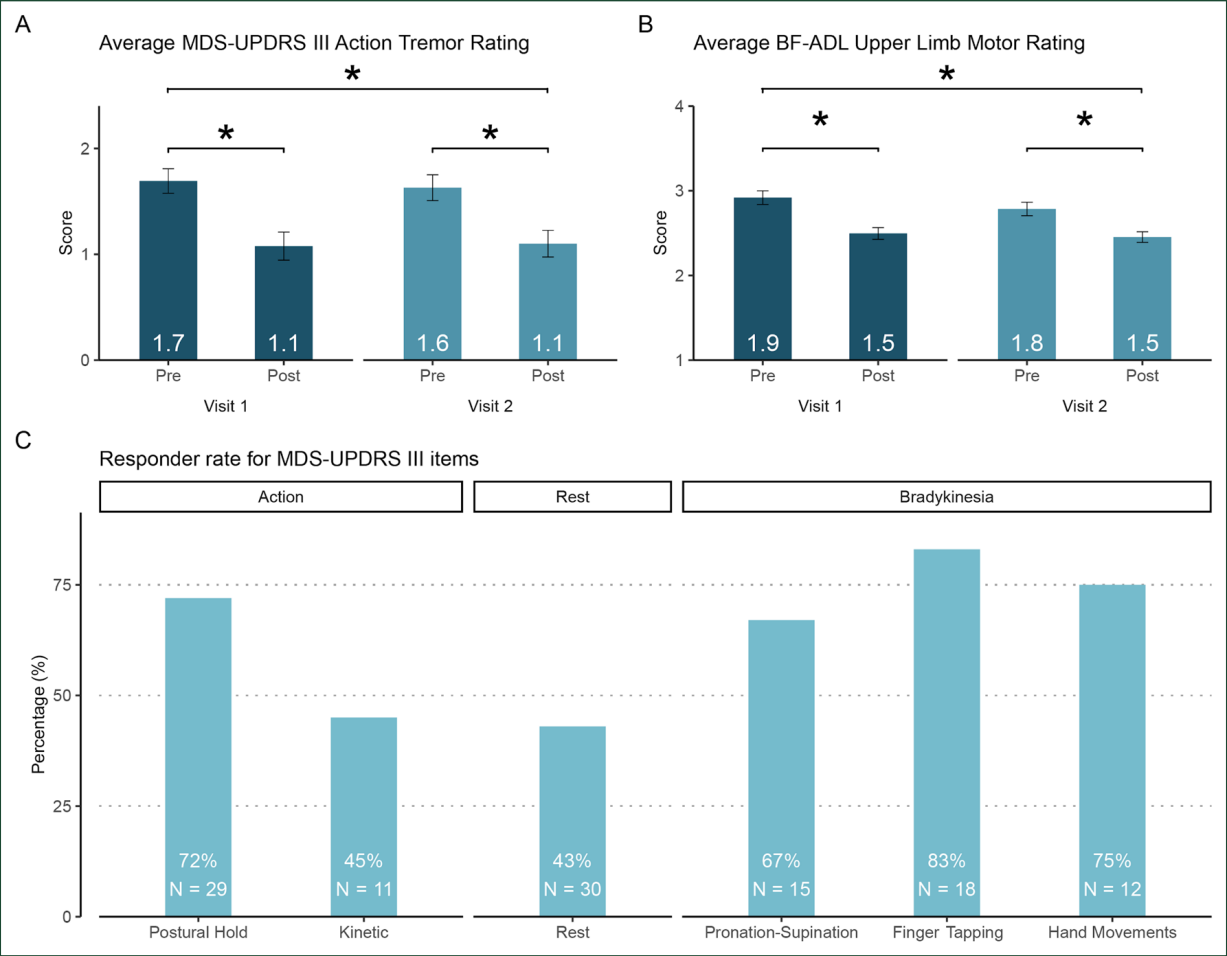
**Tremor improvement in the dominant hand while in a medication-off state during visits as rated by clinicians on MDS-UPDRS and by patients on BF-ADL (co-secondary endpoints) (Visit 1, N = 39; Visit 2, N = 35). (A)** TAPS improved clinician-ratings of average postural and kinetic tremor tasks on MDS-UPDRS (rated on a scale of 0 to 4) at each supervised stimulation session. **(B)** TAPS improved patient-ratings of average BF-ADL tasks (rated on a scale of 1 to 4) at each supervised stimulation session. **(C)** Responder rates varied across individual MDS-UPDRS III items for pre-stimulation of visit 1 to post-stimulation of visit 2. See Supplemental Table 1 and 2 for MDS-UPDRS and BF-ADL ratings across all tasks and visits. Error bars represent mean ± 1 standard error and significance (*) was tested at *p* < 0.05.

When evaluating changes from baseline to the end of the study (i.e., pre-visit 1 to post-visit 2), clinician-ratings of MDS-UPDRS III action tremor improved by 0.6 ± 0.5 (*t_(34)_* = 7.05, *p* < 0.001) and patient-ratings of BF-ADL upper limb motion improved by 0.5 ± 0.5 (*t_(34)_* =5.69; *p* < 0.001) (Figure 3A and 3B). Responder rates in MDS-UPDRS III were 72% and 45% for postural tremor and kinetic tremor respectively with rates for non-action tremor items ranging from 43% (rest tremor) to 83% (finger tapping) (Figure 3C). In BF-ADL, responder rates ranged from 52% to 88% with the 2 highest responder rates for “dial a telephone” (84%) and “insert an electric plug” (88%) (Supplemental Table S1).

MDS-UPDRS III and BF-ADL ratings also improved within both supervised stimulation sessions when comparing before to after therapy (MDS-UPDRS III action tremor visit 1 per-task improvement, 0.6 ± 0.5, *t_(38)_* = 7.80; visit 2 per-task improvement, 0.5 ± 0.5, *t_(34)_* = 6.09; BF-ADL upper limb motion ratings visit 1 per-task improvement, 0.4 ± 0.4, *t_(38)_* = 6.32; visit 2 per-task improvement, 0.3 ± 0.4, *t_(34)_* = 5.00, all *p* < 0.001; Figure 3A and 3B). Responder rates for within-visit comparison in MDS-UPDRS III and BF-ADL ranged from 36% to 78% across both visits (Supplemental Table S2).

**Figure 4:**
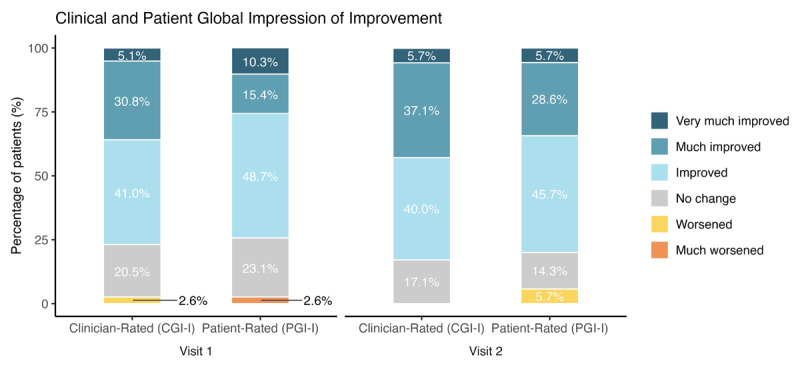
**Clinician- and patient-rated impressions of improvement during visits.** Clinicians rated motor symptoms as improved in 77.5% (visit 1) and 83% (visit 2) of patients (CGI-I) Seventy-five percent (visit 1) and 80% (visit 2) of patients self-rated improvement in motor symptoms (PGI-I) (Visit 1 N = 39, Visit 2 N = 35).

Clinicians reported improvement in 77% (36% “Much Improved” or “Very Much Improved”) and 83% (43% “Much Improved” or “Very Much Improved”) of patients on CGI-I at visits 1 and 2 respectively. Approximately three-quarters of patients reported improvement on PGI-I (26% to 34% “Much Improved” or “Very Much Improved”; Figure 4) at both visits. Most patients (78%) reported that tremor relief persisted beyond the end of stimulation, with these patients reporting a median 60-minute duration of post-stimulation relief in the exit survey.

No serious AEs were reported. Seven device-related AEs were reported from seven patients (18% AE rate). AEs on the treated hand included sore or lesion on the skin (two reports; one rated as mild severity, one moderate), persistent skin irritation (mild; subsequently diagnosed as eczema), electric shock sensation (moderate), persistent pain from stimulation (mild), and twitching of the ring finger (mild). Other AEs included development of insomnia, anxiety, and worsening of tremor at night (mild). These AEs were treated with either removal of the device or application of topical ointment and resolved within a few days without sequelae. There was one instance of gait unsteadiness, rated as a moderate severity AE. This was classified as unlikely to be device-related, but the patient discontinued study participation while awaiting assessment by their clinician.

## Discussion

TAPS provided safe and effective relief of PD-associated action tremor, similar to results of previously reported relief of ET-associated action tremor [8,9,10,11,18], suggesting TAPS may be a valuable treatment option for both PD and ET patients with action tremor. Action tremor improvement was demonstrated across multiple assessment modalities, including objective accelerometer-based motion assessments, clinician ratings and patient ratings. Furthermore, action tremor improvement was demonstrated in both a supervised clinical environment and an unsupervised home use environment, with over 1,000 remote motion-sensor measurements capturing dose-by-dose response over a month of use. The study also showed relief of other upper limb motor symptoms, including rest tremor and bradykinesia.

The efficacy of TAPS for PD patients in this study was comparable to that observed in previous ET studies. During a 3-month longitudinal study of ET-associated action tremor relief, 21,806 sessions were recorded during home use of TAPS therapy. Based on the median TPIR per patient, 92% of patients demonstrated an improvement in tremor power; 54% of patients experienced ≥50% reduction in tremor power; and 25% of patients experienced at least 70% reduction in tremor power [11]. Similarly, tremor power improvement over 1,273 sessions recorded during this 1-month study of PD- associated action tremor during home use of TAPS therapy demonstrated 97% of patients experienced improvement in tremor power; 79% of patients experienced ≥50% reduction in tremor power; and 50% of patients experienced at least 64% improvement in tremor power (Figure 2).

Efficacy in this PD study and previous ET studies both used BF-ADLs and tremor power to measure action tremor. This PD study used MDS-UPDRS III for clinical ratings while previous ET studies used Tremor Research Group’s Essential Tremor Rating Assessment Scale (TETRAS) [28]. Patients with ET experienced average improvement in BF-ADL of 0.6 points per task (mean change of 5 divided by 8 tasks) over the longitudinal home-use study [11], similar to the PD patients’ improvement in BF-ADL of 0.5 points per task in this study. While clinician-rating scales differed for the two populations, TETRAS and MDS-UPDRS III share a similar rating scale between 0 and 4. Patients with ET experienced average improvement in TETRAS upper limb performance section of 0.6 points per task within a single visit in a randomized controlled study in ET [9] and 0.5 points per task (mean change of 2.8 divided by 6 tasks) in the longitudinal home-use study [11]. These previous findings are comparable to PD patients’ average improvement of 0.6 per item in MDS-UPDRS III action tremor scores from baseline to end of the study as well as to within-visit improvement (visit 1, 0.6; visit 2, 0.5).

Adverse events were predominantly mild and resolved with minimal intervention. This study demonstrated that TAPS in PD has a side effect profile comparable to previous studies of TAPS in ET, with a device-related AE rate of 18% in this study in PD patients and 18% in the longitudinal home use clinical trial in ET patients [11]. TAPS in PD also has a more favorable side effect profile than other therapies for PD. The safety profile of TAPS was favorable to that of levodopa, whose AEs include nausea, dizziness, headache, somnolence, and motor complications that appear in about half of patients [29]. The risks with DBS, which include brain hemorrhage, stroke, infection, headache, and worsening mental or emotional status [30], are likewise considerably more severe than that of TAPS.

Several limitations of this study should be considered while interpreting its results. First, this was an open-label study. The field would benefit from a double-blinded, randomized sham-controlled study. However, the observed responder rates in postural tremor of MDS-UPDRS III (64% to 72%) were larger than 47% of PD patients who responded to placebo in tremor [31]. In addition, previous PD studies only identified a potential placebo effect in rest tremor [32,33] with one study failing to discover any therapeutic effect of transcranial magnetic stimulation for postural tremor across treatment and sham controlled groups [33]. Taken together, the postural tremor improvement observed in this study was unlikely due to a placebo effect.

Furthermore, previous studies of TAPS in ET-associated action tremor have been randomized to sham [8,9]. In the ET study including BF-ADLs and TETRAS, the magnitude of response per task to sham (0.36 and 0.35 on BF-ADL and TETRAS upper limb score) [9] was smaller than the magnitude of response per task observed in this PD study (0.3 to 0.4 and 0.5 to 0.6 on BF-ADL and MDS-UPDRS III action tremor score for within-visit improvement). The consistency in response rates between the objective accelerometer-based measures of tremor power and the clinician and patient-ratings also suggests the ratings were not strongly biased.

Second, this study controlled the timing of PD-related medication with respect to TAPS sessions during supervised stimulation sessions at study visits but not during unsupervised home use. While it is possible the tremor improvement during home use was confounded by medication dynamics, this design captured TAPS efficacy as anticipated for real-world usage. Future studies tracking the temporal dynamics and interactions between TAPS therapy and medication would provide valuable guidance to clinicians and patients on dosing TAPS therapy in patients taking PD-related medications.

Finally, this study only enrolled PD patients with postural tremor and focused on upper limb motor tasks assessable by telemedicine. Furthermore, because postural holds were performed unsupervised at home, it is possible the postural hold measurements may have unintentionally captured re-emergent tremor, in addition to postural tremor. While this enabled conducting the study during the COVID-19 pandemic, future in-person assessments on the effect of TAPS in a broader PD population and on other PD symptoms would be valuable.

## Conclusions

TAPS therapy yielded clinically meaningful improvements in action tremor in patients with PD. Adverse events occurred at a low rate and were generally mild. These findings suggest that TAPS may be a valuable treatment option for both PD and ET patients with action tremor.

## Financial Disclosures of all authors (for the preceding 12 months)

Salima Brillman M.D. Serves as a consultant for Neurocrine, Supernus, Amneal, Cala Health, Kyowa Kirin. She is on the speakers bureaus at Supernus, Amneal, Abbvie, Teva, Neurocrine, Acorda and Kyowa Kirin.

Pravin Khemani M.D. serves as a consultant for Abbott, Boston Scientific, GE, Cala Health. He is on Advisory Boards at Abbott, Boston Scientific, GE, Cala Health and receives Honoraria from GE, Cala Health, Neurocrine, Boston Scientific.

Stuart H. Isaacson M.D. receives honoraria, research grants, consults for and/or is a promotional speaker for Abbvie, Acadia, Acorda, Adamas, Addex, Affiris, Alexza, Allergan, Amarantus, Amneal, Aptinyx, Axial, Axovant, Benevolent, Bial, Biogen, Biovie, Britannia, Cadent, Cala Health, Cerecor, Cerevel, Cipla, Eli Lilly, Enterin, GE Healthcare, Global Kinetics, Impax, Impel, Intec Pharma, Ipsen, Jazz, Kyowa Kirin, Lundbeck, Merz, Michael J. Fox Foundation, Mitsubishi Tanabe, Neuralys, Neurocrine, Neuroderm, Novartis, Parkinson Study Group, Pharma2B, Praxis, Prilenia, Promentis, Revance, Roche, Sage, Sanofi, Scion, Stoparkinson, Sunovion, Sun Pharma, Supernus, Teva, Theravance, Transposon, UCB.

Rajesh Pahwa M.D. serves as a consultant for Abbott, AbbVie, ACADIA, Acorda, Amneal, Artemida, Britannia, Cala Health, Global Kinetics, Impel, Insightec, Jazz, Neuropharma, Kyowa, Neurocrine, PhotoPharmics, Sage, Scineuro, Sunovion, Supernus and XwPharma. He receives research support from Abbott, AbbVie, Addex, Biogen, Biohaven, Boston Scientific, EIP, Global Kinetics, Impax, Intec, Lilly, Neuroderm, Neuraly, Parkinson’s Foundation, Pharma 2B, Prelinia, Roche, Sage, SIS, Sun Pharma, Sunovion, Theranexus, Theravance, and Voyager.

Ruta Deshpande M.S. is a former employee of Cala Health.

Vivien Zraick M.S. is an employee of Cala Health.

Apoorva Rajagopal PhD. is a former employee of Cala Health.

Dhira Khosla D.O. is an employee of Cala Health.

Kathryn H. Rosenbluth PhD. is an employee and board member of Cala Health.

## Additional File

The additional file for this article can be found as follows:

10.5334/tohm.762.s1 Supplemental Materials. Summary of MDS-UPDRS & BF-ADL across and within visits.
