# Supplementary material for: Non-Invasive Transcutaneous Afferent Patterned Stimulation Therapy Offers Action Tremor Relief in Parkinson’s Disease
Source: Tremor Other Hyperkinet Mov (N Y). 2023 Aug 23;13:25. doi: 10.5334/tohm.762 (PMC10453948; doi:10.5334/tohm.762)
Supplement: Supplemental Materials. — Summary of MDS-UPDRS & BF-ADL across and within visits. [file tohm-13-1-762-s1.pdf]

Supplemental Materials

Table S1 Descriptive summary of MDS-UPDRS and BF-ADL for each task across visits

	Visit 1 (Month 0) Pre to Visit 2 (Month 1) Post		
Task	N[1]	Change[2]	Responder Rate
		**MDS-UPDRS**	
Postural tremor	29	-1.1 (1.0)	72%
Kinetic tremor	11	-0.5 (0.7)	45%
Rest tremor amplitude	30	-0.7 (1.3)	43%
Pronation / Supination	15	-0.7 (1.0)	67%
Finger Tapping	18	-1.0 (0.6)	83%
Hand Movements	12	-0.8 (0.8)	75%
		**BF-ADL**	
Use a spoon to drink soup	27	-0.6 (0.6)	52%
Hold a cup of tea	27	-0.6 (0.7)	59%
Pour milk from a bottle	27	-0.7 (0.7)	67%
Dial a telephone	19	-1.0 (0.6)	84%
Pick up change	15	-0.8 (0.8)	60%
Insert an electric plug	17	-1.1 (0.7)	88%
Unlock front door	16	-0.8 (0.7)	69%
Write a letter	31	-0.7 (0.8)	65%

[1] N represents the number of patients at visit 1 pre-stimulation assessment that had a rating of ≥ 2 ("Mild" impairment) on each task.

[2] Change values are reported as mean (standard deviation).

[3] % Improved is the percent of patients with impairment in assessed task that improved by ≥ 1 increment on the rating scale

Table S2 Descriptive summary of MDS-UPDRS and BF-ADL for each task within visits

	Visit 1 (Month 0)					Visit 2 (Month 1)				
Task	N[1]	Pre[2]	Post[2]	Change[2]	Responder Rate	N[1]	Pre[2]	Post[2]	Change[2]	Responder Rate
					**MDS-UPDRS**					
Postural tremor	32	2.6 (0.6)	1.7 (1.0)	-1.0 (0.9)	69%	28	2.5 (0.6)	1.6 (1.0)	-0.9 (0.9)	64%
Kinetic tremor	13	2.2 (0.6)	1.6 (0.7)	-0.5 (0.5)	54%	12	2.2 (0.4)	1.6 (0.5)	-0.6 (0.5)	58%
Rest tremor amplitude	33	3.4 (0.8)	2.9 (1.1)	-0.5 (0.8)	42%	25	3.5 (0.7)	3.0 (1.1)	-0.5 (0.7)	36%
Pronation / Supination	17	2.4 (0.5)	1.8 (1.0)	-0.6 (0.8)	53%	13	2.4 (0.5)	2.2 (0.8)	-0.2 (1.0)	38%
Finger Tapping	21	2.5 (0.5)	1.9 (1.0)	-0.6 (0.8)	38%	17	2.2 (0.4)	1.6 (0.6)	-0.6 (0.6)	59%
Hand Movements	15	2.2 (0.4)	1.7 (0.6)	-0.5 (0.6)	40%	10	2.2 (0.4)	1.3 (0.8)	-0.9 (0.7)	70%
					**BF-ADL**					
Use a spoon to drink soup	29	2.7 (0.8)	1.8 (0.8)	-0.9 (0.7)	72%	27	2.6 (0.7)	2.0 (0.7)	-0.6 (0.8)	44%
Hold a cup of tea	29	2.6 (0.7)	2.0 (0.9)	-0.6 (0.9)	55%	23	2.6 (0.8)	2.1 (0.7)	-0.5 (0.8)	39%
Pour milk from a bottle	29	2.3 (0.5)	1.6 (0.7)	-0.7 (0.8)	59%	26	2.2 (0.4)	1.8 (0.8)	-0.5 (0.8)	58%
Dial a telephone	23	2.3 (0.5)	1.4 (0.7)	-0.9 (0.7)	78%	17	2.1 (0.3)	1.4 (0.5)	-0.8 (0.6)	71%
Pick up change	18	2.2 (0.4)	1.7 (0.6)	-0.6 (0.7)	44%	13	2.2 (0.4)	1.6 (0.7)	-0.5 (0.5)	54%
Insert an electric plug	20	2.3 (0.4)	1.5 (0.6)	-0.8 (0.6)	70%	12	2.3 (0.5)	1.4 (0.7)	-0.8 (0.8)	75%
Unlock front door	17	2.2 (0.4)	1.7 (0.6)	-0.5 (0.6)	47%	14	2.1 (0.4)	1.4 (0.5)	-0.7 (0.6)	64%
Write a letter	35	2.6 (0.6)	2.0 (0.6)	-0.6 (0.6)	58%	27	2.6 (0.6)	2.0 (0.8)	-0.6 (0.6)	56%

[1] N represents the number of patients at visit that had a rating of ≥2 ("Mild" impairment) on assessed task. Note, Visits 1 and 2 included assessments of 39 and 35 patients, respectively
[2] Pre, Post, and Change values are reported as mean (standard deviation).
[3] % Improved is the percent of patients with impairment in assessed task that improved by ≥1 increment on the rating scale
Note, values provided in the Pre, Post and Change were calculated from the number of patients at visit that had a rating of ≥2 on each task.

Supplemental Methods

Tremor power data were analyzed for the primary endpoint only if no significant voluntary movement was detected during the postural holds before or after a given session. First, to avoid any disturbance caused by movement transition while performing postural hold, the initial and final 4 seconds of accelerometry data for each postural hold were removed before tremor power calculations were performed. Second, tremor

measurements were excluded if the power of accelerometry data in the voluntary movement band (0 to 4 Hz) was greater than the power in the tremor band (4 to 12 Hz), unless both the voluntary motion and tremor band power values were both less than 1 $(m/s^2)^2$. These criteria allowed for the possibility that patients could experience substantial tremor improvement, in which case tremor power could be less than voluntary movement power, and both would be less than 1 $(m/s^2)^2$.
